# Serologic Cross-Reactivity between the Mumps Virus Vaccine Genotype A Strain and the Circulating Genotype G Strain

**DOI:** 10.3390/v16091434

**Published:** 2024-09-08

**Authors:** Sabaparvin Shaikh, Michael Carpenter, Lisa Lin, Jasmine Rae Frost, Elizabeth McLachlan, Derek Stein, Paul Van Caeseele, Alberto Severini

**Affiliations:** 1 Department of Medical Microbiology and Infectious Diseases, Faculty of Health Sciences, University of Manitoba, Winnipeg, MB R3T 2N2, Canada; derek.stein@gov.mb.ca (D.S.);; 2 National Microbiology Laboratory Branch, Public Health Agency of Canada, Winnipeg, MB R3E 3R2, Canada; michael.carpenter@phac-aspc.gc.ca (M.C.); jasmine.frost@phac-aspc.gc.ca (J.R.F.); elizabeth.mclachlan@phac-aspc.gc.ca (E.M.); 3 Cadham Provincial Laboratory, Winnipeg, MB R3E 3J7, Canada

**Keywords:** mumps virus, genotype A, genotype G, antigenic variation, cross-reactivity, serology, immunofluorescence

## Abstract

Recent mumps outbreaks have been observed in vaccinated young adults due to the mumps virus (MuV) of genotype G, whereas the current vaccine is a mixture of two genotype A strains. These outbreaks could be attributed to waning vaccine immunity or the antigenic differences between the HN and F glycoproteins in the vaccine and circulating MuV. These glycoproteins are essential targets for the immune system, and antigenic variations may reduce the recognition of mumps antibodies, rendering the population susceptible to the MuV. We established stable cell lines expressing the MuV glycoproteins to study cross-reactivity between genotype A and genotype G. Cross-reactivity between the genotypes was evaluated via immunofluorescence using patient sera from vaccinated individuals, infected individuals, and vaccinated individuals infected with genotype G. Titer ratios showed that the vaccinated individuals exhibited a titer 3.68 times higher for the HN protein and 2.3 times higher for the F protein when comparing genotype A with genotype G. In contrast, the infected individuals showed a lower titer for genotype A compared with genotype G, at 0.43 and 0.33 for the HN and F proteins, respectively. No difference in titer ratio was observed for individuals vaccinated and subsequently infected with mumps. These findings suggest that antigenic variations between the two genotypes may potentially result in immune escape of the circulating strain, resulting in individuals susceptible to the MuV.

## 1. Introduction

The mumps virus (MuV), a member of the paramyxovirus family, genus *Rubulavirus*, is a negative-strand RNA virus with a 15,384 nucleotide genome [1]. It is transmitted through respiratory droplets, particularly in closed areas with prolonged contact, including small communities, sports teams, and university campuses [1,2]. The incubation period is 16–18 days post-exposure [2]. A significant portion (30–50%) of MuV infections are asymptomatic or result in mild symptoms, including fever, headaches, muscle aches, and swelling of the salivary glands, especially the parotid glands [1,2]. These mild symptoms usually occur in young children infected with mumps, and they tend to recover within a week [3]. More severe complications, such as inflammation of the testes (orchitis), ovaries (oophoritis), pancreas (pancreatitis), and brain and spinal cord (meningitis), as well as deafness, have been reported in adults, with the frequency of these complications being lower in vaccinated individuals [3].

The MuV genome contains seven linked transcription units, which include the four core proteins: the virion/phosphoprotein (V/P), large protein (L), nucleoprotein (NP), and the matrix protein (M); two glycoproteins, the hemagglutinin-neuraminidase (HN) and the fusion (F) protein; and the membrane-associated small hydrophobic protein (SH) [2,4,5]. The two glycoproteins, HN and F, play an essential role in viral entry into the host cell and are targets for the host immune system [6,7,8]. The roles of the MuV HN and F proteins have been reviewed previously by Frost et al. (2022) [9].

The MuV is considered a monotypic virus, and so far, 12 genotypes from A to N (excluding E and M) have been defined based on the SH gene RNA sequence [10,11]. Prevaccination mumps had an incidence rate of 1/1000, and the introduction in Canada and the US of the MMR vaccine in 1971 decreased the caseload by 99% [12]. In North America, the Jeryl Lynn (JL) vaccine, a live attenuated vaccine containing a mixture of JL5 and JL2 strains belonging to genotype A, is used [13]. The mumps vaccine is available as a trivalent vaccine, MMR (measles, mumps, and rubella), or a quadrivalent vaccine, MMRV (measles, mumps, rubella, and varicella) [13]. Two doses of the vaccine are given, the first at 12–15 months and the second at 4–6 years of age [13].

Since 2006, several mumps outbreaks caused by the genotype G MuV strains have been observed in highly vaccinated populations [14,15,16]. These outbreaks could be due to waning vaccine immunity or antigenic variations between the vaccine genotype A strains and the circulating genotype G strains [14,15,16]. The differences between the HN and F proteins of genotype A and genotype G have been recently reviewed [9]. For the HN protein, genetic variations produce changes in the protein N-glycosylation sites, membrane anchorage domain, and B- and T-cell epitopes [9]. For the F protein, the variations lead to changes in the signal peptide regions and in the carboxy terminal region [9]. Antigenic variations between genotype A and genotype G could result in a lack of cross-reactivity in vaccine-induced antibodies and potential immune escape [9,14,15,16].

Enzyme immunoassays (EIA) and plaque reduction neutralization tests (PRNT) have been used to study mumps putative antigenic variations between genotype A and genotype G [17,18,19,20,21,22,23,24,25]. Studies using EIA to measure total antibodies against mumps have shown no correlation in antibody titer in individuals vaccinated for mumps compared with individuals infected with mumps [17,18,19,20,21]. Several studies using PRNT to measure neutralizing antibodies have only shown that vaccine-induced antibodies can neutralize the genotype G strains, but the level of neutralization of genotype G is lower than for genotype A [22,23,24,25]. In contrast, several other studies and unpublished results from our laboratory have found no correlation between the MuV genotype A and genotype G PRNT results [24,25,26]. EIA-based assays are limited by the amount of antigen and the kits used, and variations in PRNT results may occur from variability within viral strains, viral titer, and infectivity resulting in a lack of reproducibility across studies. Some studies have suggested that the immune response to mumps may be strain-specific rather than genotype-specific, and the neutralization efficacies may depend on the evolutionary divergence of the strains [26,27]. Overall, it is unclear if differences in the immune response toward the MuV vaccine strains are significant, and a better approach is needed to study the cross-reactivity between mumps genotype A and genotype G [28].

To detect more reliably if possible antigenic variation between genotype A and genotype G could leave vaccinated individuals susceptible to mumps, we devised an immunofluorescence assay (IFA) using cell lines expressing the glycoproteins HN and F. IFA is a very sensitive test that detects antibodies against proteins expressed in cells in their native conformation. Because of their sensitivity and specificity, IFA tests are considered the gold standard for a number of infections, for example, the Epstein–Barr virus serodiagnosis [29,30,31]. Antibody titers are determined by a limiting dilution series, and the last dilution at which a signal is obtained is noted as the titer for serum. IFA analysis is independent of the antigen concentration as long as the proteins are expressed in excess of their cognate antibodies and, therefore, it is not biased toward possible differences in expression between proteins from different mumps strains [31].

Using IFA, we compared sera from vaccinated individuals without prior infection of genotype G, unvaccinated individuals with genotype G infection, and individuals vaccinated and subsequently infected with the MuV genotype G.

## 2. Materials and Methods

### 2.1. Lentivirus Plasmids Cloning

Whole genome sequencing (WGS) was performed for both the genotype A reference strain (VE-12343) and the genotype G CDC strain, and sequences were analyzed using Molecular Evolutionary Genetics Analysis (MEGA) using methods previously published [32]. The HN and F protein sequences were codon-optimized, and restriction enzyme sites and Kozak sequence were added to the 5′ end of the DNA (Table S1). A FLAG epitope tag was added at the 3′ end of the HN and F DNA sequences. The HN and F protein gene sequences from each genotype were commercially ordered in a puc17 vector (GenScript, Piscataway, New Jersey, USA and Thermofisher, Waltham, Massachusetts, USA). PCR amplification was performed using gene-specific primers for the HN and F proteins of genotype A and genotype G with the SuperFii enzyme, using the company suggested protocol (Thermofisher, cat#12358010). The resulting PCR fragments were purified using a QIAGEN PCR purification kit (Qiagen, cat#28104, Hilden, Germany).

The lentivirus vector pCDH-CMV-Neo containing the CMV promoter and the neomycin resistance gene were obtained from System Biosciences (cat#CD514B-1). The vector and the PCR products were digested with Xba1 (NEB, cat#R0145S, Ipswich, Massachusetts, USA) and Not1 (NEB, cat#R0189S) restriction enzymes for 1 h at 37 °C. The digested vector and PCR products were run on a 0.7% agarose gel and gel-purified using the QIAEX II gel purification kit (Qiagen, cat#20021). A ligation reaction was performed overnight at 16 °C using NEB T4 DNA ligase (NEB, cat#B0202S) to ligate the vector and insert at a 1:3 ratio.

The ligation products were used to transform One Shot TOP 10 *E. coli* cells (Invitrogen, cat#C4040-3, Shanghai, China) that were plated on LB-Carbenicillin plates, followed by overnight incubation at 37 °C. Isolated colonies were grown overnight in 5 mL LB broth with ampicillin, and plasmid DNA were isolated using the QIAprep Miniprep kit (Qiagen, cat#27104). Restriction digestion with MfeI (NEB, cat#R3589S) and EcoRI (NEB, cat#R0101S) and agarose gel analysis were performed to ensure that the fragment sizes were correct. The correct clones were then sent to the National Microbiology Laboratory (NML) DNA core for NGS sequencing to ensure no mutations were present in the plasmid. Once verified, plasmid DNA were isolated for transfection using the Qiagen Midi prep Plus kit (QIAGEN, cat#12943).

### 2.2. Lentivirus Generation and Transduction

293TN cells were used for transfections and maintained in DMEM medium supplemented with 4 mM L-glutamine, 4.5 g/L glucose, 10% FBS, and 100 units/mL of Penicillin/Streptomycin. To generate lentivirus, 2 × 10^6^ cells/mL were seeded in a 10 cm plate for 24 h to achieve 70–80% confluency. For transfection, 1.7 µg of cloned lentivirus plasmid expressing the HN and F proteins or vectors only, 16 µL of packaging mix containing 2.8 µg of gag and rev plasmids, and 2.7 µg of VSV-G plasmid were added to 1 mL of Opti-MEM media. Post transfection, 30 µL of Roche X-tremeGene HP (cat#6366244001, Basel, Switzerland) was added and incubated for 15 min at room temperature and added dropwise to the cells. After 24 h, fresh medium was added to the cells. At 48 h post-transfection, cells were examined under a microscope to confirm adherence, and the supernatant was collected and filtered through a 45 µm cellulose filter. To concentrate the virus, 2.5 mL of PEG-IT reagent (System Biosciences, cat#LV810A-1, Palo Alto, California, USA) was added to the collected supernatant, gently mixed, and stored overnight at 4 °C. Subsequently, the virus and PEG-IT mixture were centrifuged at 1500× *g* for 40 min at 4 °C, and the resulting virus pellet was resuspended in 1 mL of PBS then stored at −80 °C.

Vero cells were used to express the MuV glycoproteins and maintained in MEM medium supplemented with 5% FBS (complete medium). Fifty thousand cells were seeded to each well of a 24-well plate and incubated overnight at 37 °C with 5% FBS to achieve a 50% confluency. For lentivirus transduction, growth media were removed and 100 µL of lentivirus, 97 µL of complete media, and 3.2 µL of a 0.5 mg/mL polybrene solution (Sigma, cat#H9268-5G, St. Louis, Missouri, USA) were added to the cells. The plate was incubated with occasional rocking for seven hours in the incubator. After the transduction period, the mixture was removed, and 1 mL of fresh medium was added to the cells. After 24 h, the cells were washed once with medium, and then fresh medium was added to maintain their viability. At 72 h post-transduction, antibiotic selection was applied for two weeks by supplementing complete medium with 700 µg/mL of Geneticin (G418) (Thermofisher, cat# 10131-027). Following the antibiotic selection, the cells were maintained in MEM medium with 5% FBS and 500 µg/mL of G418 to sustain the transduced cell population.

### 2.3. Immunofluorescence

An immunofluorescence assay was used to confirm protein expression and antibody binding differences in stable cell lines. Thirty thousand Vero cells expressing the MuV glycoproteins and vector control were seeded in 8-well chamber slides (Thermofisher, cat#177402) in complete medium with antibiotics and incubated at 37 °C in 5% CO_2_.

The cells were washed once with PBS and fixed with 4% paraformaldehyde for 10 min. After fixation, the cells were washed three times with PBS for 5 min each and permeabilized with a 0.5% Triton X solution diluted in PBS for 5 min. Subsequently, the cells were washed three times with PBS for 5 min and blocked for 1 h with 1X BSA in TBS.

Cadham Provincial Laboratory (Manitoba, Canada) kindly provided residual mumps diagnostic sera from vaccinated individuals (Genotype A, n = 19), infected individuals (Genotype G, n = 9), and individuals vaccinated and infected (Genotype A and Genotype G, n = 10) (Table S2). The cells were stained overnight with goat anti-mouse Flag antibody (Thermofisher, cat# MA1-91878) at a dilution of 1:300 in 1% BSA in TBST, which was used to confirm the expression of the transgene glycoproteins. A 2-fold dilution series ranging from 1:32 to 1:4012 was also performed using the patient serum.

After the overnight incubation, cells were washed three times with PBS and incubated for 1 h with goat anti-mouse AF488 antibodies (Thermofisher, cat# A55058) at 1:1500 and goat anti-human AF555 (Thermofisher, cat#A-21433) at 1:2000 dilution in 1% BSA diluted in TBST, in the dark. The cells were then washed thrice with PBS and incubated with DAPI (1:37000) (Thermofisher, cat# D21490) for 5 min. The cells were washed with PBS once, and fresh PBS was added. The cells were then imaged with the EVOS FL Color Imaging system under the DAPI, GFP, and RFP channels. The obtained images were analyzed, and the lowest dilution at which a signal was observed was recorded as per the methodology for serum titer, as is I confirmconducted in EBV diagnostics [30,31]. The titer was scored by 3 independent evaluators and any discrepancies between the evaluators were resolved by a 4th evaluator. The geometric mean titer (GMT) with a 95% confidence interval was calculated using the serum titer for the three groups as the titer data are not normally distributed. Titer ratios were calculated by dividing genotype A over genotype G, and the arithmetic mean with a 95% confidence interval for the three groups, as the ratios are independent of positively skewed results. A limitation of the study methods is that no technical replicates were performed due to restricted serum volumes.

## 3. Results

### 3.1. Antigenic Variations between MuV Genotype A and Genotype G Strains

MEGA was used to identify amino acid (aa) variations in the MuV HN and F proteins between the genotype A vaccine strain and genotype G circulating strain. For the MuV HN protein, 31 aa variations were observed between genotypes A and G (Table 1). These variations resulted in changes in the N-glycosylation sites, membrane anchorage domain, predicted escape neutralization sites, and known B- and T-cell epitopes [33,34,35,36]. Additionally, 16 aa variations sites were unique and have not been reported in the literature; some of the variations are present in known neutralization escape sites and may result in immune escape [35].

For the MuV F protein, 28 aa variations were observed between the genotype A and genotype G strains (Table 2). The variations resulted in changes in the signal peptide regions and in carboxy terminal regions [35]. Notably, 20 aa variations were unique between genotype A and genotype G and have not been reported in the literature.

### 3.2. Generation of Stable Cell Lines Expressing MuV Glycoproteins

**Figure 1:**
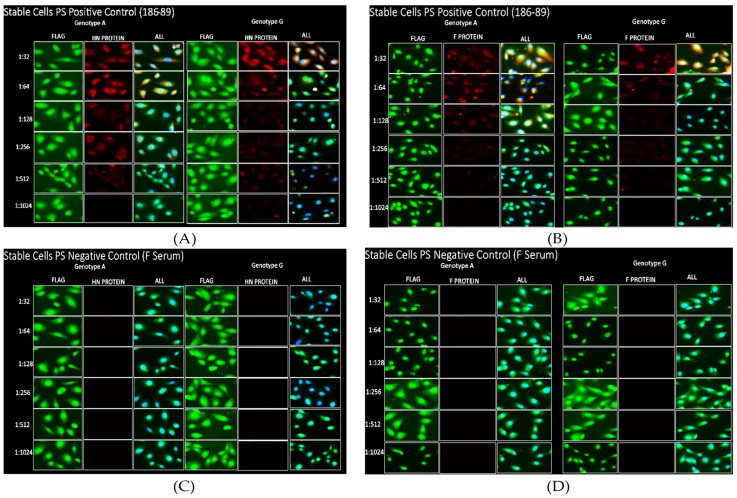
(**A**) Stable cell lines expressing the HN protein incubated with the positive control serum 186-89. (**B**) Stable cell lines expressing the F protein incubated with the positive control serum 186-89. The serum 186-89 was used as positive control as it had a high ND_50_ for PRNT and the titers for this serum were read as positive at a 512 and at a 1024 fold dilution for HN protein genotype A and genotype G, respectively. For the F proteins, the titers were read as positive at a 512 fold dilution for both genotype A and genotype G. (**C**) Stable cells negative control serum F for HN protein. (**D**) Stable cells negative control serum F for F protein. Serum F was used as a serum-negative control as it was negative for the MuV antibody by ELISA and BioPlex 2200 and showed no signal (antibody binding) for the HN and F proteins. FLAG antibody (FLAG) was used without anti-mumps serum to check for protein expression. ‘All’ contains DAPI, the green channel (FLAG), and the red channel (HN Protein) all merged together.

Lentivirus clones were generated using codon-optimized HN and F DNA sequences using the pCDH-CMV-Neo vector. No mutations were observed in the cloned vector, hence lentiviruses were generated and used to express the MuV HN and F proteins from genotype A and genotype G in Vero cells. Positive selection was performed using a gentamycin antibiotic, and all the live cells post-selection expressed the HN and F proteins. Positive detection with an anti-FLAG antibody demonstrated that the HN and F proteins were expressed (Figure S1). The in-house positive control serum 186-89, for which the infection or vaccination history is not available, showed a positive MuV antibody response by ELISA and was confirmed by PRNT). Serum 186-89 showed a strong IFA signal at the highest dilution for both the HN and F proteins for both genotypes and a decrease in signal with serial dilution, as expected (Figure 1A,B). The signal from the FLAG tag and the positive control colocalized (Figure 1A,B). Serum F, a serum from an individual with no history of mumps vaccination or infection, was used as a negative control and was negative for the mumps antibody by ELISA and the Bioplex 2200 MMRV IgG (Alltech Inc, Lexington, Kentucky, USA) assay. “Strip-serum”, a commercial serum stripped of IgG antibodies, was also used as a negative control. Both serum F and the “strip-serum” showed no nonspecific binding for the HN and F proteins (Figure 1C,D). Taken together, these results show that the stable cell lines were expressing the MuV HN and F glycoproteins, and an IFA system allowed us to detect anti-HN and anti-F antibodies in a sensitive and specific manner. A limiting dilution series was performed and the last dilution at which a signal was observed was noted as the titer.

### 3.3. Cross-Reactivity of Mumps Genotype A and Genotype G Antibodies

**Figure 2:**
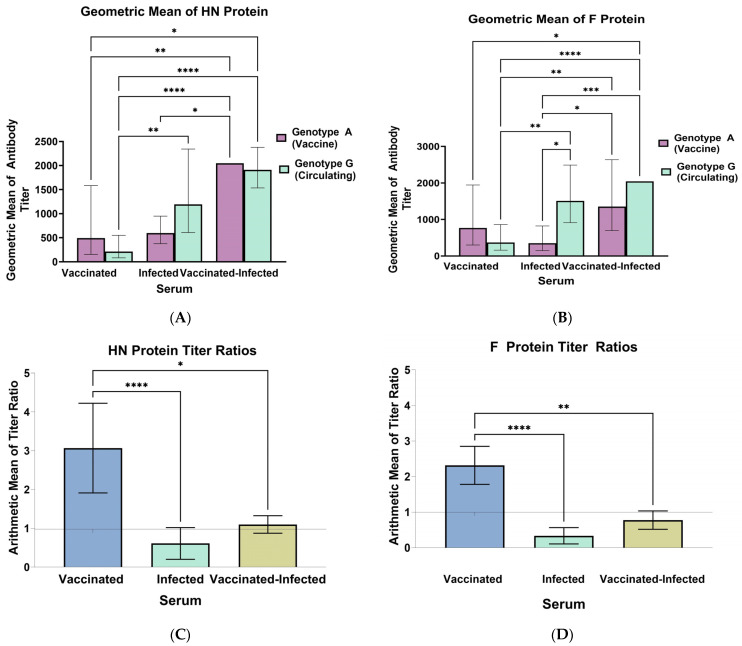
(**A**) GMT for the HN protein antibodies. (**B**) GMT for the F protein antibodies. (**C**) Titer ratio for the HN protein. (**D**) Titer ratio for the F protein. Titer ratios were calculated by dividing genotype A over genotype G (A/G). The vaccinated group includes individuals vaccinated for mumps (n = 19). The infected group includes individuals infected with mumps and no history of vaccination (n = 9). The vaccinated –infected group includes individuals vaccinated for mumps and subsequently infected with MuV genotype G strains (n = 10). The error bars represent the 95% confidence intervals. Statistical significance is presented as *p*-value < 0.05 represented by *, *p*-value < 0.01 is represented by **, and *p*-value < 0.0002 is represented by ***, and *p*-value <0.0001 ****.

The stable cell lines expressing the MuV HN and F glycoproteins were used to study the cross-reactivity of mumps antibodies, using serum from vaccinated, infected, and vaccinated–infected individuals. The serum titer was measured for the three different groups for both HN and F proteins, from A and G genotypes, and the GMT was calculated (individual values are shown in Tables S3 and S4). The 95% confidence interval and the Kruskal–Wallis test with the post hoc Dunn’s test were conducted for the multiple comparisons. For the anti-HN antibodies, the GMT was 592 for genotype A and 213 for genotype G for vaccinated individuals (Figure 2A). For infected individuals, the genotype A titer was 645, and the genotype G titer was 1505 (Figure 2A). For vaccinated–infected individuals, the genotype A and genotype G titer were both 2048 (Figure 2A). The results showed that the vaccinated individuals had a significantly lower GMT for genotype G compared to infected and vaccinated–infected individuals.

For the anti-F antibodies, the GMT was 765 for genotype A and 369 for genotype G for vaccinated individuals (Figure 2B). For the infected individuals, statistically significant differences were observed between genotype A, which had a GMT of 348, and genotype G, which had a GMT of 1505 (Figure 2B). For vaccinated –infected individuals, the GMT was 1351 and 1910 for genotype A and genotype G, respectively (Figure 2B). Statistically significant differences were also observed in the genotype G titer for vaccinated individuals compared to individuals that had been infected and vaccinated–infected.

For the HN and F proteins, titer ratios were calculated by dividing genotype A over genotype G (A/G) to normalize the results for immune response variance between different groups. For the anti-HN antibodies, in the vaccinated group, the titer ratio was 3.68, and for the anti-F antibodies, the titer ratio was 2.3 (Figure 2C,D). The titer ratio for infected individuals was 0.43 for anti-HN antibodies and 0.33 for anti-F antibodies (Figure 2C,D). For vaccinated–infected individuals, the titer ratio was 1 for anti-HN antibodies and 0.80 for anti-F antibodies (Figure 2C,D). The 95% confidence intervals were calculated, and significant differences were observed between all the groups except the infected and vaccinated–infected groups for the HN and F proteins (Figure 2C,D).

## 4. Discussion

The results show that differences in cross-reactivity exist between mumps genotype A and genotype G and may be attributed to antigenic variations in the MuV glycoproteins. Antigenic variations between the vaccine genotype A strains of the MuV and the circulating genotype G strains could result in reduced recognition of vaccine-induced antibodies for genotype G, resulting in individuals being clinically susceptible to wild-type mumps. Amino acid (aa) variations between the genotype A reference strain (VE-12343) and genotype G (CDC strain) were compared, and 31 aa variations were observed for the HN protein and 28 aa variations for the F protein.

For the HN protein, 15 aa variations were reported in the literature, and 16 aa were unique, not reported before in genotype G, although five of the variations (aa25, aa121, aa122, aa123, and aa442) have been reported in genotypes F, H, and I circulating in Asia and South America [9,11,24,33,34,35,36,37]. Variations in the HN protein resulted in changes in the N-glycosylation sites, membrane anchorage domain, and known predicted B- and T-cell epitopes [9,33,34,35,36]. The H464N amino acid variation caused structural changes in the HN protein by replacing a bulky uncharged amino acid with a smaller charged amino acid. The H464N variation also added an additional N-glycosylation site in the HN protein. [38]. Studies have shown variations in aa80, aa81, aa279, and aa287 present in the B- and T-cell epitopes sites, resulting in immune escape due to reduced HLA binding and a mismatch in CD4 and CD8 T-cell response [35,36]. The variations at glycosylation sites and in known epitopes results in structural changes and increased hydrogen bonds, and the addition of charged amino acids can potential result in strong protein binding, increasing the viral infectivity and reducing recognition of neutralizing antibodies [5,33,36]. Variations in aa356 and aa464 have resulted in increased neurovirulence but the exact mechanism remains to be studied [36]. B- and T-cell epitopes predictions studies have identified several peptides for the MuV HN protein variations that result in neutralization escapes, and the presence of these mutations could reduce the recognition of neutralizing bodies, resulting in differences in cross-reactivity between the mumps genotype A and genotype G.

For the F protein, 28 aa variations were observed, and 20 aa variations were unique. Of the 20 aa variations that are unique for genotype G, 4 aa variations are found in wild-type genotype A (SBL-1, Enders, and Kilham Strains), C, and D strains [9,37,38]. Some of the variations are found in the signal peptide region and the carboxy terminal region, but the effect of these changes has not been studied, and further studies are needed to determine the effect of variations on the function of the F protein [35,36,37].

We developed an IFA system to detect differences in antibody binding between genotype A and genotype G glycoproteins in mumps vaccinated, infected, and vaccinated–infected individuals. For both the HN and F proteins, the results show that vaccinated individuals had a higher antibody titer for genotype A than for genotype G, and the reverse was observed for individuals who had not been vaccinated but were infected with the MuV genotype G. For vaccinated–infected individuals, there were no differences in genotype A and genotype G titers for both the HN and F proteins. These results show that differences in cross-reactivity exist for both genotype A and genotype G, and vaccinated individuals may not be protected against circulating genotype G strains.

These results are in agreement with previous work that measured mumps antibody response in children and in mouse models and observed neutralization capacities that differed between genotype A and genotype G [36,39]. Antigenic variations between genotype A and genotype G could result in decreased neutralizing titer observed in genotype G [35,36]. Santak et al. (2006) studied neutralization differences using sera from mice immunized with genotype A vaccine strains and the genotype G Dutch strain using PRNT and observed that variation from the vaccine strain result in decreased neutralization capacity [36]. Rubin et al. (2008) found that the GMT for the genotype A was twice as that of genotype G in vaccinated populations [40]. Rasheed et al. (2019) also performed PRNT and observed a six-fold decrease in the GMT for the wild-type genotype G strain compared to the genotype A vaccine strain [40]. The studies, combined with our results, show that the antigenic differences between genotype A and genotype G result in reduced titer against genotype G, potentially due to antigenic differences or a more pronounced waning immunity against genotype G.

Studies looking at the correlate of protection for mumps by EIA-based assays and PRNT have found no correlation [41,42,43,44]. EIA-based assays measure total antibodies whereas neutralization-based assays measure neutralizing antibodies, which could result in a lack of correlation. Poor correlation could also be due to differences in viral strains, titer, and infectivity, as there are a lack of standards available [45,46,47]. To obviate these differences, we decided to use an IFA system to look at differences in antibody titer between the genotype A and genotype G HN and F proteins as the majority of the neutralizing antibodies are produced against the two glycoproteins. The IFA system is independent of an antigen concentration and is easy to reproduce. Our results show that there is a difference in cross-reactivity between the mumps genotype A and genotype G, however, due to the lack of correlate of protection it is difficult to conclude whether these differences leave individuals susceptible to mumps. Nevertheless, these results show that differences in cross-reactivity exist, and thus a new vaccine could be needed to counteract differing immunity in young adults.

A limitation of the study is that the time post-infection of serum sample collection relative to the disease was not available and if the serum samples for infected and vaccinated–infected were collected during an active mumps infection, a higher antibody response would have been observed. A larger sample size would allow us to increase the statistical power of the observed results and provide further validation of the results.

## 5. Conclusions

The goal of the study was to generate stable cell lines expressing the MuV HN and F proteins to study cross-reactivity between mumps genotype A and genotype G, using IFA analysis. Stable cell lines present the HN and the F proteins in their native conformation, and the IFA analysis is largely independent of the amount of protein, which is in excess of their cognate antibodies. Therefore, differences in antigen preparations between A and G genotypes do not affect the calculation of the endpoint dilution titer. Titer results show there is a partial lack of cross-reactivity between mumps genotype A and genotype G for the HN and F proteins. However, since there is no correlate of protection known for mumps, it is difficult to conclude if these differences predict an individual’s susceptibility to mumps.

## Figures and Tables

**Table 1:** Amino acid variation in the MuV HN protein between genotype A and genotype G.

Position	Amino Acid Variation	Potential Effect	Literature Reference or Unique
6	L6F	N/A	[35]
8	I8T	N/A	Unique
9	M9I	N/A	[35]
12	N12S	Loss of N-Glycosylation	[36]
21	V21S	N/A	[35]
25	G25N	N/A	Unique
37	V37A	Change in Membrane Anchorage Domain	Unique
44	I44T	Change in Membrane Anchorage Domain	Unique
56	I56V	Change in Membrane Anchorage Domain	[35]
80	A80T	Change in Membrane Anchorage Domain and T-cell Epitope	[34]
81	V81M	Change in Membrane Anchorage Domain and T-cell Epitope	[34]
113	S113A	N/A	Unique
121	N121S	Neutralization Escape *	Unique
122	R122Q	Neutralization Escape *	Unique
123	N123K	Neutralization Escape *	Unique
135	I135V	N/A	Unique
218	A218V	N/A	Unique
279	I279T	Change in B-cell and T-cell Epitope	[33,36]
287	I287V	Change in B-cell and T-cell Epitope	[33,36]
356	E356D	Change in B-cell Epitope	[33,36]
372	S372N	N/A	[33,36]
375	V375I	Neutralization Escape *	[35]
399	N399S	Neutralization Escape *	[35]
403	L403M	N/A	Unique
442	Y442S	Neutralization Escape *	Unique
444	Q444P	N/A	Unique
464	H464N	Addition of N-glycosylation	[35]
473	I473T	N/A	Unique
474	V474T	N/A	Unique
490	R490S	N/A	Unique
577	A577T	N/A	[35]

* Variations at these sites have resulted in neutralization escape in some genotype A strains and may cause neutralization escape in genotype G strains [9,35].

**Table 2:** Amino acid variation in the MuV F protein between genotype A and genotype G.

Position	Amino Acid Variation	Potential Effect	Literature Reference or Unique
2	N2K	Change in Signal Peptide Region	[35]
3	A3V	Change in Signal Peptide Region	[35]
4	F4S	Change in Signal Peptide Region	[35]
5	P5L	Change in Signal Peptide Region	Unique
7	I7T	Change in Signal Peptide Region	[35]
11	Y11F	Change in Signal Peptide Region	Unique
13	I13V	Change in Signal Peptide Region	[35]
16	S16F	Change in Signal Peptide Region	[35]
24	T24I	N/A	Unique
49	V49I	N/A	Unique
95	L95P	N/A	Unique
170	D170N	N/A	Unique
177	S177N	N/A	Unique
269	M269V	N/A	Unique
318	R318S	N/A	Unique
330	H330N	N/A	Unique
331	M331I	N/A	Unique
345	S345T	N/A	Unique
409	S409A	N/A	Unique
413	L413V	N/A	[35]
431	T431A	N/A	Unique
454	S454I	N/A	[35]
477	F477V	N/A	Unique
479	V479I	N/A	Unique
480	G480N	N/A	Unique
488	V488I	Change in Carboxy Terminal	Unique
489	S489A	Change in Carboxy Terminal	Unique
498	I498M	Change in Carboxy Terminal	Unique

## Data Availability

Available on request.

## Supplementary Materials

The following supporting information can be downloaded at: https://www.mdpi.com/article/10.3390/v16091434/s1, Table S1: Primer sequence for PCR amplification. Extra nucleotides and restriction sites are underlined; Table S2: List of primary antibody serum for immunofluorescence microscopy; Table S3: Serum titer for the MuV HN protein; Table S4: Serum titer for the MuV F protein; Figure S1: Stable cells negative control for the HN and F proteins.
