# Supplementary material for: Serologic Cross-Reactivity between the Mumps Virus Vaccine Genotype A Strain and the Circulating Genotype G Strain
Source: Viruses. 2024 Sep 8;16(9):1434. doi: 10.3390/v16091434 (PMC11437446; doi:10.3390/v16091434)
Supplement: Supplementary file 1 [file viruses-16-01434-s001.zip › viruses-3146367-supplementary.pdf]

Supplementary File:

Table S1. Primer sequence for PCR amplification. Extra nucleotides and restriction sites are underlined. Capital Letters include the start of the gene sequence.

Protein Target	Genotype	Primer	Sequence (5′ to 3′)
HN	A	Forward	cacatctagagccaccATGGAACCGTCAAAGTT
HN	G	Forward	cacatctagagccaccATGGAACCATGGAACCTAGC
F	A	Forward	cacatctagagccaccATGAACGCTTTTCCAG
F	G	Forward	cacatctagagccaccATGAAGGTGTCTCTGG
HN & F	A & G	Reverse	gtgtgcgcccgcTCACTTGTCATCATCGTCCT

Table S2. List of Primary Antibody Serum for Immunofluorescence Microscopy.

Antibody/Patient Serum	Use	Number of Samples	Dilution Series
F Serum	Negative Control	N/A	1:32-1:4012
Strip Serum	Negative Control	N/A	1:32
186-89 Serum	Positive Control	N/A	1:32-1:4012
Mumps Vaccinated Only	Test Serum	19	1:32-4012
Mumps Infected Only	Test Serum	9	1:32-4012
Mumps Vaccinated/Infected	Test Serum	10	1:32-4012

Table S3. Serum Titer for the MuV HN protein.

Sample Number	Vaccinated		Infected		Vaccinated-Infected	
	A	G	A	G	A	G
1	2048	512	512	1024	2048	2048
2	2048	1024	512	2048	2048	2048
3	512	128	256	512	2048	2048
4	2048	1024	1024	2048	2048	2048
5	2048	256	2048	2048	2048	2048
6	512	256	512	2048	2048	2048
7	512	256	1024	2048	2048	2048
8	512	128	1024	2048	2048	2048
9	2048	256	512	1024	2048	2048
10	512	512			2048	2048
11	512	256				
12	512	128				
13	2048	512				
14	256	128				
15	256	128				
16	64	32				
17	256	128				
18	256	32				
19	256	256				

Table S4. Serum Titer for the MuV F protein.

Sample Number	Vaccinated		Infected		Vaccinated-Infected	
	A	G	A	G	A	G
1	512	128	256	2048	2048	2048
2	256	256	256	2048	2048	2048
3	256	128	512	512	2048	2048
4	1024	512	64	1024	2048	2048
5	64	64	512	2048	512	2048
6	512	128	512	1024	2048	2048
7	256	128	512	2048	2048	2048
8	512	256	128	2048	2048	2048
9	1024	512	1024	2048	512	2048
10	2048	512			512	1024
11	1024	512				
12	2048	1024				
13	2048	1024				
14	1024	512				
15	1024	1024				
16	1024	1024				
17	2048	512				
18	2048	512				
19	1024	512				

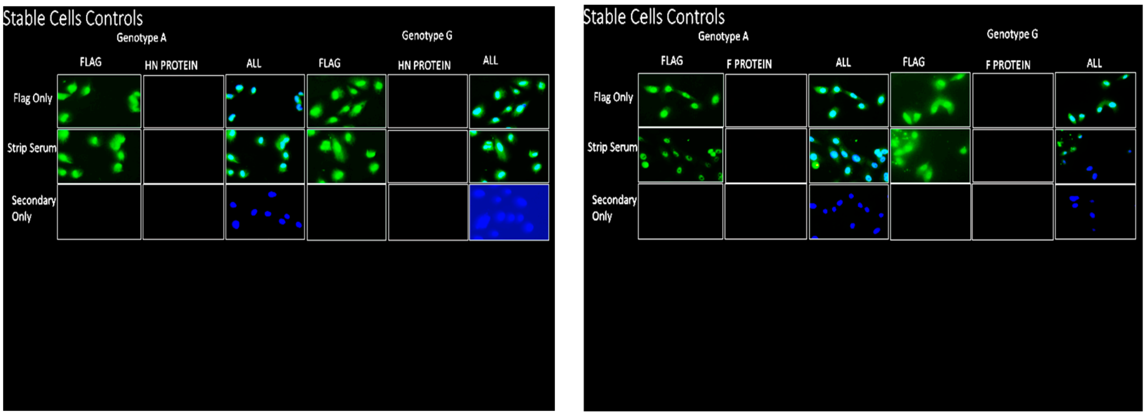

(**a**) (**b**)

Figure S1: (a) Stable Cells Negative Controls for HN protein; (b) Stable Cells Negative Controls for F Protein. Flag antibody only was used to control for bleeding between channels. Strip serum was used at a 1:32 dilution; the secondary antibody only was used as serum control. No non-specific binding was observed for the HN and F proteins. 'All' contains DAPI, the green channel (flag), and the red channel (HN Protein) all merged together.
